# Life history traits and reproductive performance of the caridean shrimp *Lysmata boggessi*, a heavily traded invertebrate in the marine aquarium industry

**DOI:** 10.7717/peerj.8231

**Published:** 2020-01-23

**Authors:** Michael D. Dickson, Donald C. Behringer, J. Antonio Baeza

**Affiliations:** 1 Fisheries and Aquatic Sciences, University of Florida, Gainesville, FL, USA; 2 Emerging Pathogens Institute, University of Florida, Gainesville, FL, USA; 3 Department of Biological Sciences, Clemson University, Clemson, SC, USA; 4 Smithsonian Marine Station at Fort Pierce, Fort Pierce, FL, USA; 5 Departamento de Biología Marina/Facultad de Ciencias del Mar, Universidad Católica del Norte, Coquimbo, Chile

**Keywords:** *Lysmata*, Population dynamics, Marine life, Caridean, Protandric simultaneous hermaphroditism

## Abstract

The most intense commercial harvest of marine aquarium species in North America occurs in the coastal waters surrounding Florida, yet very often little information exists on the life histories, population dynamics, or reproductive characteristics of these organisms. The peppermint shrimp *Lysmata boggessi* is one such species and is heavily targeted along the west coast of Florida. It is known primarily among aquarists for its ability to control pest anemones and in the scientific community for its unique sexual system, protandric simultaneous hermaphroditism. However, no study has addressed fishery interactions or long-term population dynamics for *L. boggessi*. We used monthly fisheries-dependent sampling, with a trained observer present, for a full year to assess seasonality in sex phase ratio (males to males + hermaphrodites), size at sex change, fecundity, embryo volume and reproductive output of an exploited *L. boggessi* population. *L. boggessi* exhibited distinct seasonality in size distribution, sex phase ratio, size at sex phase change and reproductive activity. The peak reproductive season was in spring, when the population was dominated by small but fecund hermaphrodites. Reproduction decreased during fall and winter and sex phase ratios favored male phase shrimp that exhibited delayed sex change. This population and individual level information is the first of its kind for *L. boggessi* and fills a much needed data gap for the informed management of this fishery.

## Introduction

Caridean shrimps (infraorder Caridea) belonging to the genus *Lysmata*, Risso 1816, are heavily targeted in the marine ornamental trade (Calado et al., 2017; Baeza & Behringer, 2017; Rhyne et al., 2017) and have experienced increases in market demand (Rhyne et al., 2009). Many marine ornamentals provide needed ecosystem services in home aquariums, such as algal–grazing and scavenging and are a biological alternative to other mechanical and chemical methods for aquarium maintenance (Calado et al., 2003; Rhyne et al., 2009). In the United States, Florida is the center of the ornamental fishery (Larkin et al., 2001), where *Lysmata boggessi* and *Lysmata wurdemanni* are collected as bycatch between October and May from commercial stone crab traps and are landed year-round in the commercial bait shrimp trawl fishery (Baeza & Behringer, 2017; Rhyne & Lin, 2006). These shrimp are desired among aquarists specifically for their ability to regulate the pest anemone *Aiptasia* spp., Gosse 1858 (Rhyne, Lin & Deal, 2004), which is often introduced to aquaria via live rock. Previous studies suggest that their recent increase in popularity is in part driven by this biological control (Rhyne et al., 2009).

The overall increase in landings of marine invertebrates that provide ecosystem services has raised concerns regarding the impact of harvest on wild stocks and their surrounding environment (Adams, Larkin & Lee, 2001; Bolker et al., 2002; Rhyne et al., 2009; Baeza & Behringer, 2017). For instance, Bolker et al. (2002) found that many ornamental fisheries operated on small spatial scales and that harvest could result in a nearly 50% localized reduction in population size. Unfortunately, there are severe data gaps in the life history, reproductive biology, population structure and growth characteristics for many of these organisms (Calado et al., 2003; Rhyne et al., 2009), which is especially true for ornamental crustaceans and *Lysmata* spp. The alarming result is that ornamental fisheries in Florida have not been managed using fisheries data and no management strategies currently inform the catch targets (i.e., maximum sustainable yield, total allowable catch) necessary to ensure stock sustainability (Bolker et al., 2002). If these data gaps were to lead to fisheries mismanagement and the overexploitation of ecosystem service providers such as *Lysmata*, then an unintended ecological consequence would be the loss of those same services from the wild (Rhyne et al., 2009; Baeza & Behringer, 2017).

In addition to their fishery and ecosystem value, shrimp from the genus *Lysmata* are notable for their rare sexual system, protandric simultaneous hermaphroditism (PSH). Individuals displaying PSH settle and mature initially as males and over a series of transitional molts develop functional female gonads to become simultaneous hermaphrodites (Fielder et al., 2010; Zhang & Lin, 2005). The latter sex phase, characterized by the presence of ovotestes and gonopores (Bauer & Holt, 1998) is capable of functioning as either sex but cannot self-fertilize (Bauer, 2000). Phylogenetic studies have determined that PSH is a fixed and conserved trait within the genus (Baeza, 2009, 2010a; Baeza & Fuentes, 2013), which is thought to be advantageous for increasing mating opportunities in low density populations and fitness specifically for large male phase shrimp (Baeza, 2007, 2010b; Lin & Zhang, 2001). Interestingly, the size at which *L. wurdemanni* males transition to hermaphrodite varies with season and appears flexible, which is suspected to be a strategy to decrease reproductive output when conditions are unfavorable (Baeza & Bauer, 2004; Baldwin & Bauer, 2003). If true across the genus, this adaptive capability may be an important consideration in the management of other harvested populations.

Baeza et al. (2014) produced a snapshot of the life history parameters of a Florida *L. boggessi* population, but they did not capture the important detailed seasonality to these parameters. We implemented a similar methodology as used by Baeza et al. (2014) and Baldwin & Bauer (2003) to assess life history and reproductive characteristics, but focused on a fished population of *L. boggessi* on the west Florida shelf. These shrimp are found in nearshore waters and are landed year round as bycatch in the commercial bait shrimp (*Farfantepenaeus duorarum*) industry. The primary objective was to describe the seasonality of sex phase ratio and size at sex change at the population level and fecundity, embryo volume and reproductive investment at the individual level.

## Materials and Methods

### Study site, sampling protocol and shrimp measurements

**Figure 1:**
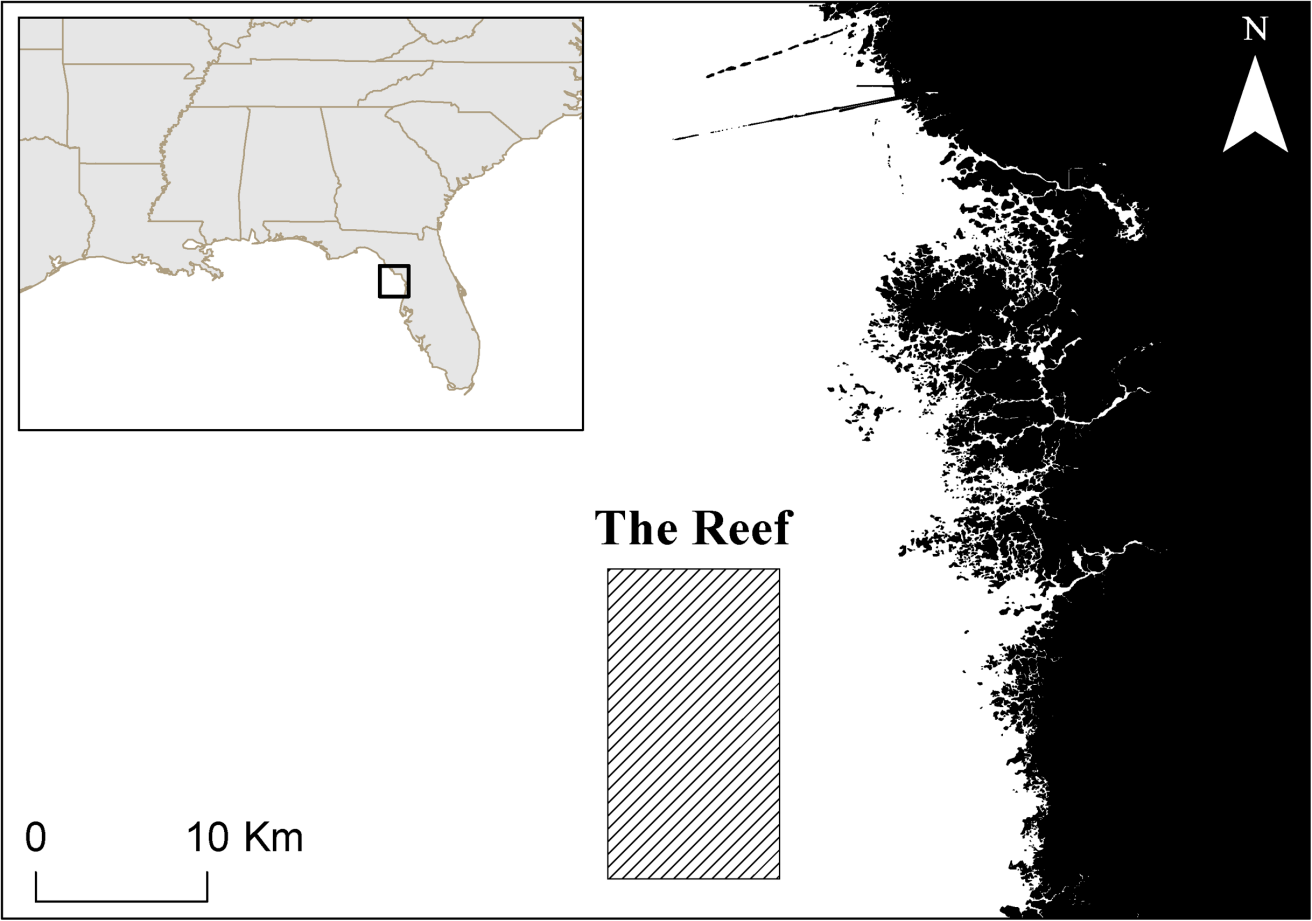
The study area, known as The Reef, which is heavily targeted for live bait shrimp (*Farfantepenaeus duorarum*) and ornamental species.

We sampled a segment of the *L. boggessi* population on the Florida west coast using fisheries-dependent techniques for a full year. This genetically homogeneous population ranges from Key West to approximately Cedar Key, Florida (Baeza & Behringer, 2017). Shrimp were collected by a trained observer at night and as bycatch once per month from December 2012 to November 2013 via roller-frame trawlers (Berekeley, Pybas & Campos, 1985) in a shallow subtidal region off the west coast of Florida. This area, locally referred to as The Reef, is located adjacent to the St. Martin’s Aquatic Preserve, 6–8 km offshore of Citrus County, Florida (Fig. 1). The depth ranged from 2 to 5 m and the benthic substrate was composed of heterogeneous coarse sand, seagrass and low relief hard bottom areas. Seagrass areas were dominated by *Thalassia testudinum* and *Syringodium filiforme* and the hard bottom was characterized by exposed limestone covered with a thin (<2 cm) layer of sediment. *L. boggessi* shelter diurnally within crevices found in hard bottom (Baeza et al., 2014), but their cryptic and nocturnal nature poses a logistical challenge for collecting specimens. We were able to circumvent this limitation by using commercial trawlers, which provided sufficient catch efficiency to achieve the sample sizes required for a statistically robust population assessment. Nocturnal segregation of shrimps by size or ontogenetic phase was not suspected in *L. boggessi* based on previous population studies of *L. wurdemanni* (Baldwin & Bauer, 2003). Therefore, we are confident that the haphazard trawler coverages collected representative samples of *L. boggessi* on The Reef.

During each sampling event the trawl vessel simultaneously deployed two roller-frame trawls (4.27 m height × 0.61 m width), one on the port and one on the starboard side, 8–10 times between sunset and 02:30 h. Trawl durations were 30–45 min. A mesh size of 25.4 mm was used near the mouth of the trawl net and throughout the tapered body, with a finer mesh catch bag (19.1 mm) woven into the tailing end. The vessel tracks, average speeds and tow times were recorded with a hand-held Garmin^™^ GPS Map 60CSX. *L. boggessi* landed during each deployment were counted and approximately 30 individuals on alternating deployments were haphazardly sub-sampled for further measurements back at the laboratory. The sub-sampled shrimp were fixed onboard the fishing vessel in 10% neutral buffered formalin and were transferred after 48 h to 70% ethanol for preservation.

Preserved specimens were viewed under a Leica^®^ Model S8AP0 dissecting stereomicroscope to measure body size, determine sex phase and assess embryonic development in gravid hermaphrodites. Carapace length (CL) was measured using Leica^®^ Application Suite V4 image analysis software to 0.1 mm from the mid-dorsal posterior margin of the carapace to the posterior edge of the eye orbital. Sex phase was determined by observing the second pair of pleopods for either the presence or absence of the appendices masculinae, where presence indicated male phase and absence indicated hermaphroditic phase (Bauer & Holt, 1998). Hermaphrodites were considered gravid if they retained an embryonic mass on the ventral side of their abdomen. Masses were delicately removed with forceps and the embryos were then counted at 10× power under the stereomicroscope to determine batch fecundity. We were not able to confidently categorize embryo maturation based on eyespot and yolk sac development alone, so we used only early embryo masses void of either feature for this analysis. Therefore, fecundity estimates were not corrected for embryo loss. To calculate embryo volume, 10 embryos were haphazardly sub-sampled from each removed mass and measured along their short and long axes to a precision of 0.001 mm. Lastly, hermaphrodites and their corresponding embryo mass were dried at 60 °C for 24 h and weighed separately to approximate reproductive output.

### Population-level life history parameters in *Lysmata boggessi*

Temporal differences in sex phase ratio, in terms of the proportion of male phase shrimp and size at sex change (CL_50_) were assessed from the monthly sub-samples. Sex phase ratio was estimated as the number of male phase individuals divided by the total number of males plus hermaphrodites in the population during each month (Baeza et al., 2010). CL_50_ was calculated via binomial logistic regression with 95% confidence interval bounds. The CL_50_ estimates represented the CL at which *L*. *boggessi* would have a 50% probability of being either male phase or hermaphroditic, with the likelihood of hermaphroditism increasing as CL exceeded the CL_50_ point estimate (Baeza et al., 2014).

### Individual-level reproductive characteristics in *Lysmata boggessi*

For hermaphrodites with early stage embryo masses, fecundity was determined as the number of embryos per individual. Reproductive investment was calculated as the quotient dry embryo mass divided by its respective dry hermaphrodite mass and represents the resource allocation of an individual towards reproduction (Baeza et al., 2014). For embryos that were removed for measurement, their volume was calculated with the formula (Turner & Lawrence, 1979):

$${\rm EV }= \frac{1}{6} ({LS}^{2}{\rm \pi})$$

where *L* denotes the long axis measurement and *S* the short axis. The mean volume estimate for an individual hermaphrodite was the average of the 10 sub-sampled embryos.

**Figure 2:**
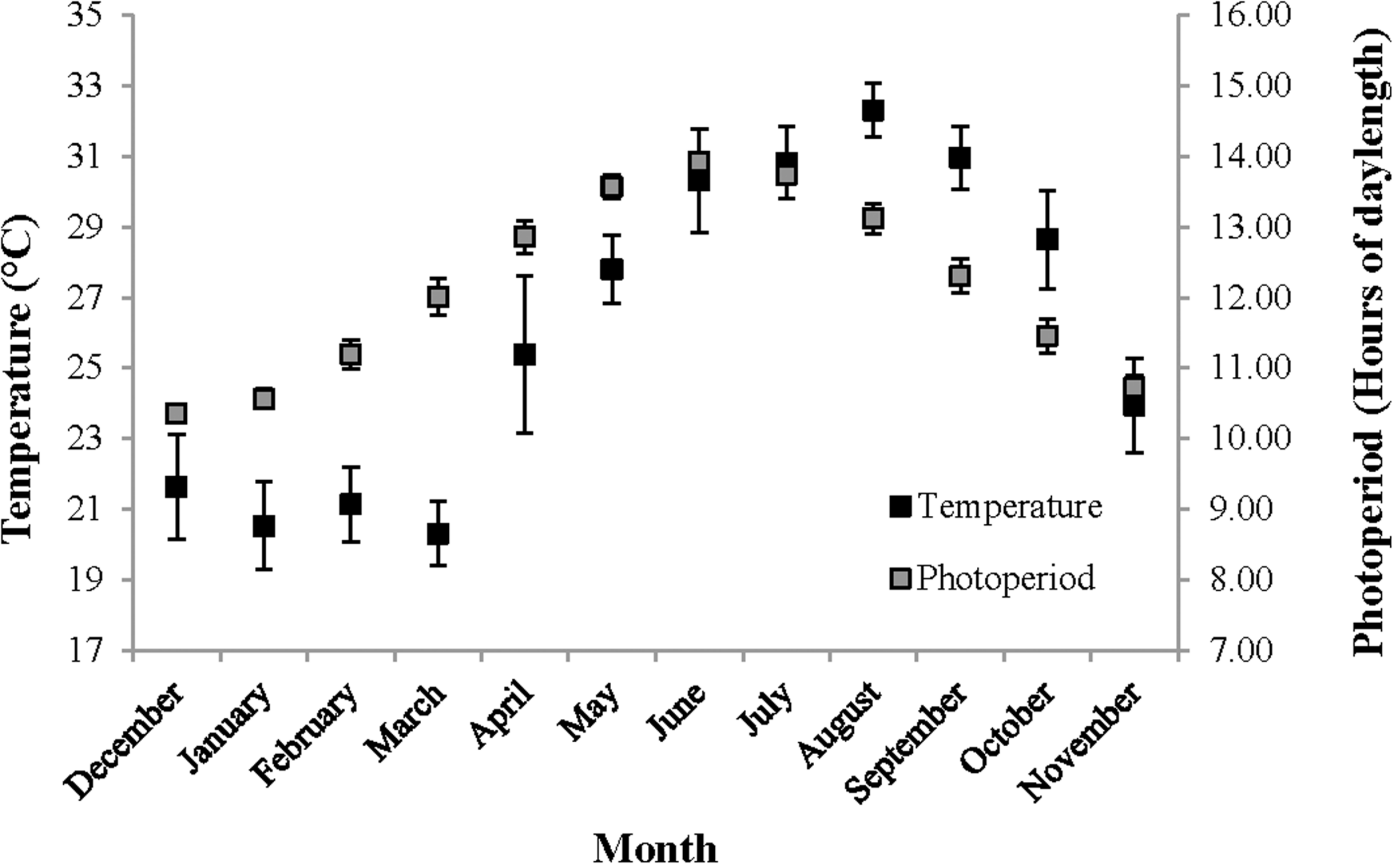
Temperature and photoperiod readings representative of The Reef. Temperature and photoperiod readings, representative of The Reef, recorded for an entire year beginning on 1st December 2012. Temperature (°C) was measured by the National Ocean and Atmospheric Association (NOAA) in nearshore waters off of Tampa, Florida, and is shown using the primary *Y*-axis. Daily photoperiod readings were taken in Orlando, Florida and are shown the secondary *Y*-axis. These temperature and photoperiod measurements were recorded within the same coastal region and at the approximate latitude, respectively, as Homosassa, Florida.

For the following seasonal analyses, the categorization of months into seasons was based on local temperature readings and photoperiod oscillations representative of the study area (Fig. 2). Hereafter, December–February is referred to as winter, March–May as spring, June–August as summer and September–November as fall.

For fecundity and embryo volume, their relationships with CL and hermaphrodite body mass, respectively, were fit with general linear models and their slopes tested (*t*-test) to determine if they differed significantly from zero. Analyses of covariance (ANCOVA) were used to determine the effect of the primary factor (i.e., season) on reproductive output, fecundity and embryo volume. Monthly samples were pooled by season to achieve adequate sample sizes and only individuals with early stage embryos were used for the reproduction analyses. Only one covariate, either hermaphrodite body mass or CL, was controlled for in each independent analysis. The linear relationship between reproductive investment and hermaphrodite dry body mass was also tested and assessed using the allometric model *y = ax^b^*, where *b* indicates the exponential increase or decrease of reproductive allocation per unit of shrimp body mass. A log–log transformed least-squares regression of the variables reproductive output and hermaphrodite dry body mass was analyzed using a *t*-test to determine if the slope deviated from expected unity (*b* = 1) (Hartnoll, 1982). Significance for all tests was achieved at α = 0.05.

## Results

### Landings of *Lysmata boggessi*

**Figure 3:**
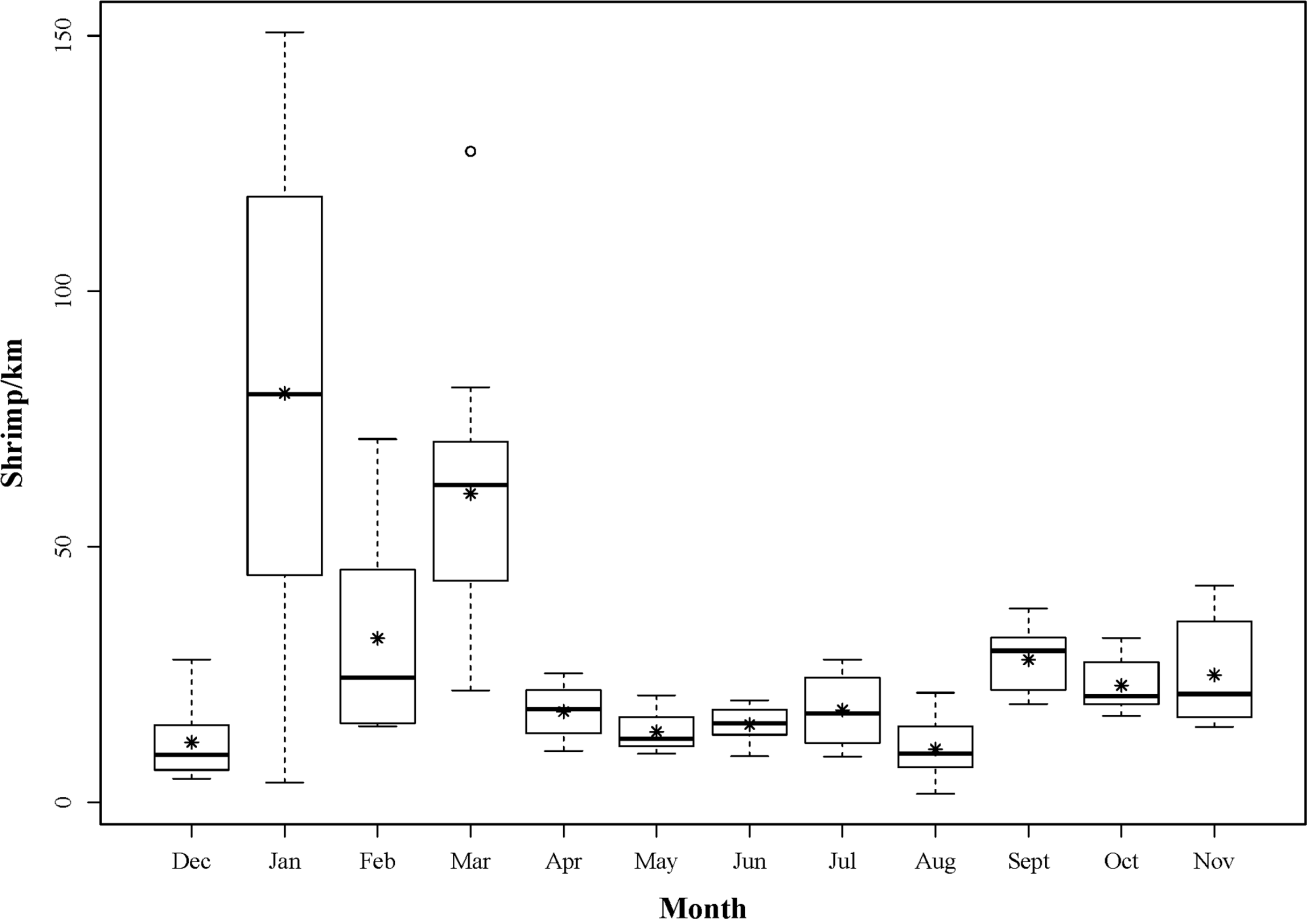
Boxplot showing the monthly distributions of shrimp abundance, calculated for each tow as the number of shrimp landed divided by the trawl distance (km). The asterisks represent mean abundance.

Sampling commenced on 6th December 2012 and concluded on 7th November 2013. Although intra-month variability in landings could not be accounted for due to the nature of scheduling fisheries dependent sampling, monthly sampling was achieved with a median number of 31 days between each sampling event. A total of 5,131 shrimp were landed over this study period (Fig. 3). Each month, 118 ± 10 (mean ± 1 SD) individuals were sub-sampled for additional detailed measurements. CLs ranged from 3.9 to 11.3 mm and 776 individuals were determined to be male phase. Of the remaining 638 hermaphrodites, 498 were gravid at the time of capture.

### Population-level life history parameters in *Lysmata boggessi*

**Figure 4:**
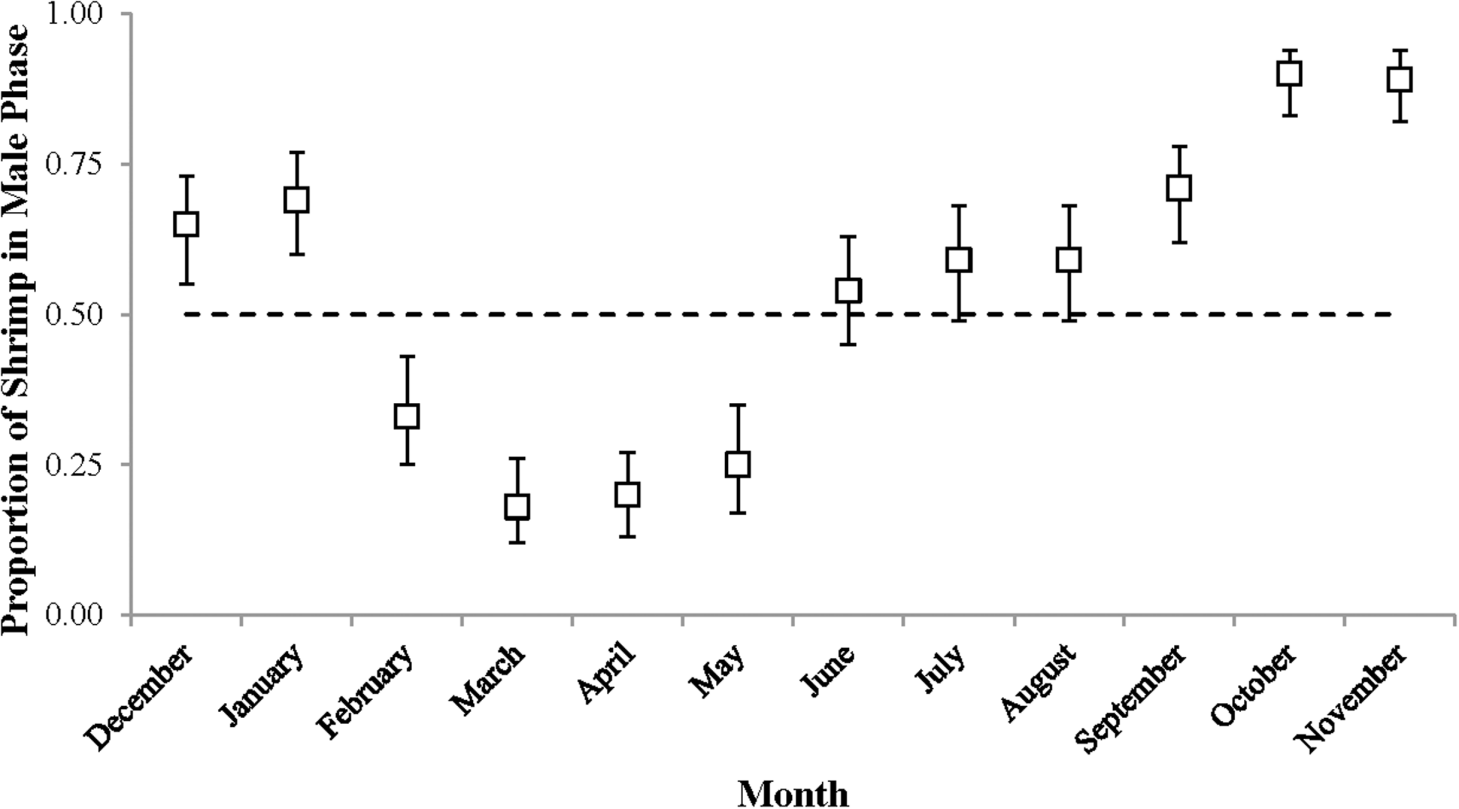
The proportion of shrimp in male phase (±1 SE) for each monthly sample. These proportions indicate the sex ratio. The dashed line represents a 1:1 male: hermaphrodite ratio.

Sex phase ratio varied between 0.18 in March and 0.90 in October with a mean of 0.54 ± 0.24, *n* = 12. Variation was plotted against a dashed line representing a 1:1 male: hermaphrodite ratio (Fig. 4). The population was skewed toward males from September to January (0.65–0.90) and toward hermaphrodites from February to May (male proportions 0.18–0.33).

**Figure 5:**
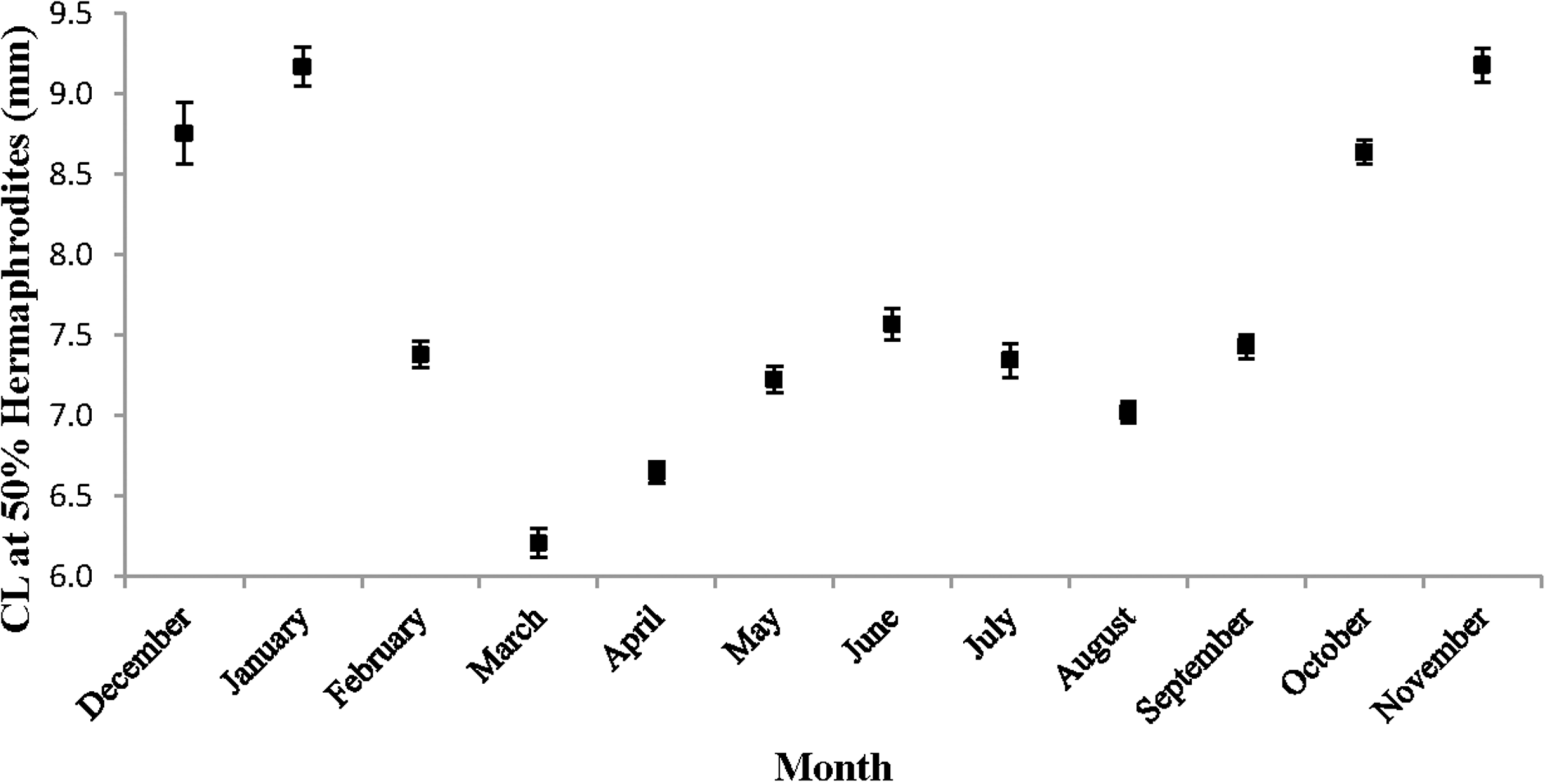
Monthly carapace length (±1 SD) at 50% hermaphroditism. Individuals at this point had a 50% probability of having already undergone sex change. The probability of sex change increases and decreases for carapace length measurements above and below this estimate, respectively.

**Figure 6:**
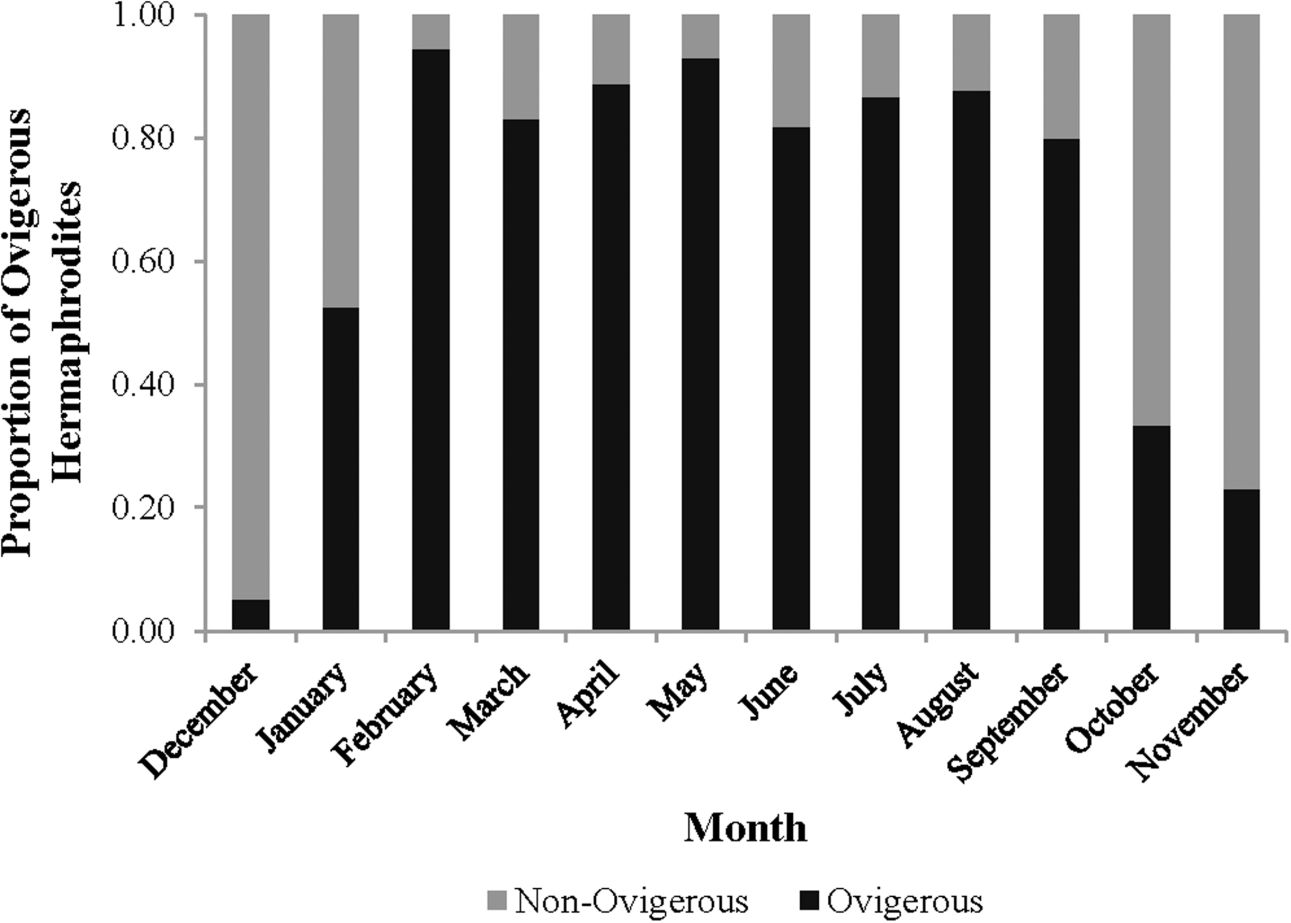
Monthly proportion of ovigerous vs. non-ovigerous hermaphrodites.

The monthly CL_50_ was highly variable and ranged from 6.20 ± 0.09 mm in March to 9.17 ± 0.12 mm in January (Fig. 5). The proportion of ovigerous hermaphrodites ranged from 0.05 in December 2012 to 0.94 in February 2013, with the highest proportions occurring from February to May (Fig. 6). These proportions remained consistently high (>0.50) throughout the year, except during October, November and December, when they were less than 0.40. Generally, high proportions of ovigerous hermaphrodites coincided with low CL_50_ estimates.

### Individual-level reproductive characteristics in *Lysmata boggessi*

**Figure 7:**
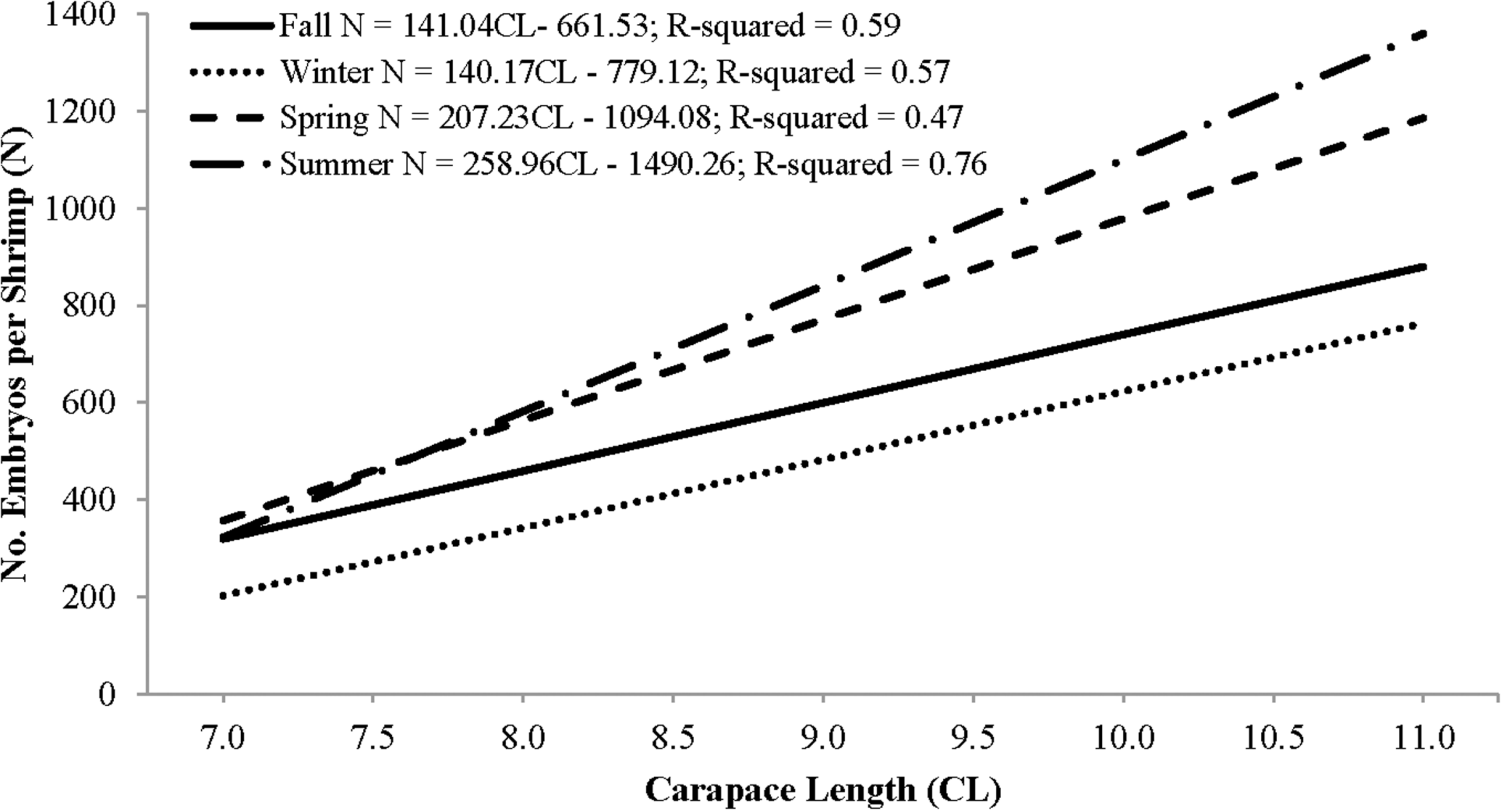
Seasonal regressions showing the relationships between the number of embryos per shrimp (i.e., fecundity) and carapace length.

**Figure 8:**
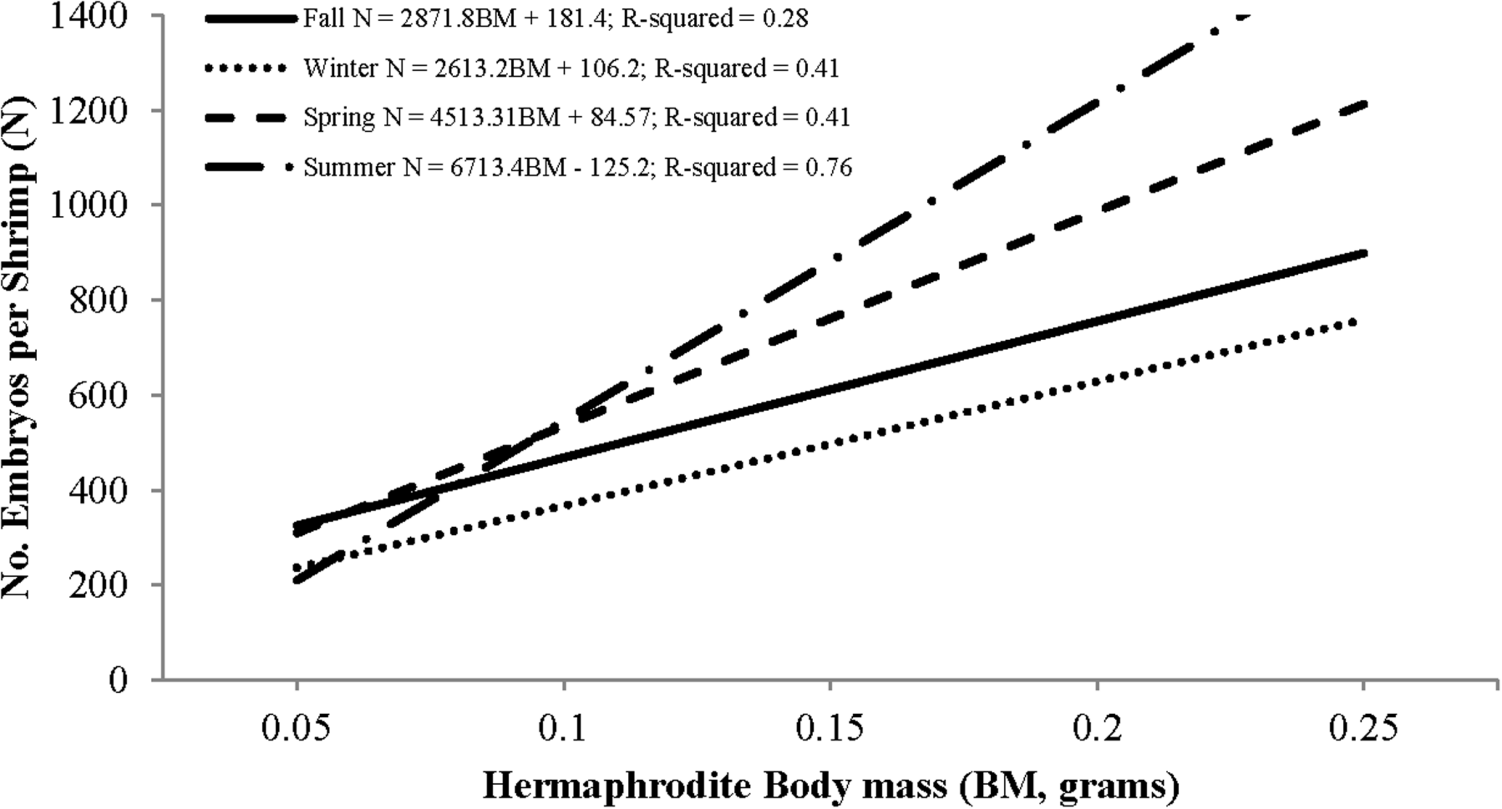
Seasonal regressions showing the relationships between the number of embryos per shrimp (i.e., fecundity) and dry hermaphrodite body mass.

Individual fecundity ranged from 45 to 1,614 embryos per shrimp, 642 ± 293 (mean ± 1 SD) and was highly variable among seasons and hermaphrodite sizes. All seasonal fecundities were positively correlated with CL and hermaphrodite body mass and mean embryos reached a high in spring and a low in fall (Table 1; Figs. 7 and 8). The two ANCOVA analyses used to test the effect of season on fecundity revealed strong seasonality. The covariates of CL and hermaphrodite body mass both resulted in significant interaction effects (*p*-values < 0.001), indicating that the relationship between each covariate and fecundity differed between seasons. Posthoc Tukey’s multiple comparison tests showed that the spring and summer fecundities were greater than fall, spring was greater than summer and summer was greater than winter (*p*-values < 0.001).

**Table 1:** Seasonal fecundity estimates and regression parameters for the relationships between the dependent variable fecundity and both independent variables carapace length and hermaphrodite dry body mass.

Season	*n*	Mean (SD)	CL parameters			Body mass parameters		
			Slope (SE)	Intercept (SE)	*R*^2^	Slope (SE)	Intercept (SE)	*R*^2^
Fall	25	472 (137)	141.0 (24.3)*	−662 (196)	0.59	2,871 (946)	181 (98)	0.25
Winter	69	474 (160)	140.1 (14.8)*	−779 (133)	0.57	2,613 (380)	106 (55)	0.4
Spring	158	748 (293)	207.2 (17.6)*	−1,094 (157)	0.47	4,513 (4,340)	84 (66)	0.41
Summer	80	700 (337)	259.0 (16.3)*	−1,490 (139)	0.76	6,713 (421)	−125 (54)	0.76

**Note:**

* Significant at α = 0.05.

Embryo volume ranged from 0.056 to 0.230 mm^3^, with winter having the highest mean of 0.15 ± 0.02 mm^3^, *n* = 69 and summer having the lowest mean of 0.10 ± 0.01 mm^3^, *n* = 80. Other than a slightly positive, but significant, correlation between volume and hermaphrodite body mass during winter, there were no statistically significant relationships between embryo volume and either CL or body mass (Table 2). The results of the ANCOVA showed an effect of season on embryo volume (*p*-value < 0.001), though the interaction term was not significant (*p*-value = 0.10) and therefore the interaction term was removed and the ANCOVA analyses re-run. This second test produced similar main effect outcomes. Posthoc Tukey multiple comparison tests showed that during winter hermaphrodites produced larger embryos than hermaphrodites in any other season (Table 2). Spring hermaphrodites produced the second largest embryos and no significant difference in volume was found between summer and fall.

**Table 2:** Seasonal embryo volume estimates and regression parameters for the relationships between the dependent variable embryo volume and independent variables carapace length and hermaphrodite dry body mass.

Season	*n*	Mean (SD)	CL parameters			Body mass parameters		
			Slope (SE)	Intercept (SE)	*R*^2^	Slope (SE)	Intercept (SE)	*R*^2^
Fall	25	0.11 (0.01)	0.0007 (–)	0.06 (0.04)	0.03	0.23 (0.14)	0.09 (0.01)	0.58
Winter	69	0.15 (0.02)	0.0007 (–)*	0.09 (0.03)	0.04	0.22 (0.07)	0.12 (0.01)	0.57
Spring	158	0.13 (0.02)	0.0001 (–)	0.14 (0.01)	0.0003	0.02 (0.04)	0.13 (0.01)	0.47
Summer	80	0.10 (0.01)	0.0002 (–)	0.09 (0.01)	0.01	0.09 (0.04)	0.09 (0.01)	0.76

**Note:**

* Significant at α = 0.05.

**Figure 9:**
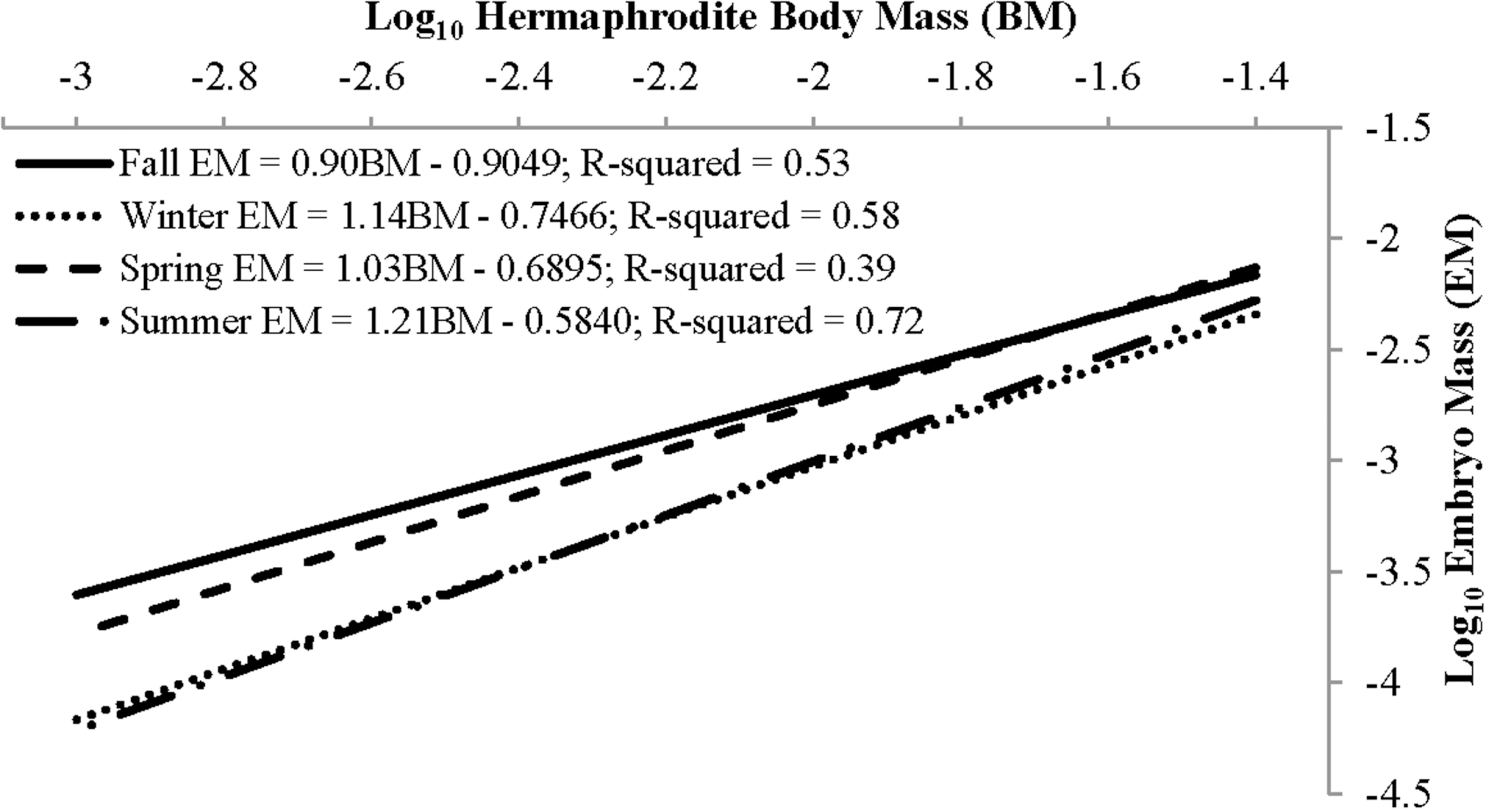
Seasonal reproductive output as the linear relationship (after log–log data transformation) between embryo mass and dry hermaphrodite body mass.

The mean reproductive investment ranged from 0.14 ± 0.04, *n* = 69, in winter to 0.20 ± 0.05, *n* = 158, in spring (Table 3). In all seasons other than summer, reproductive investment increased linearly with hermaphrodite body mass, as the slope describing the relationship between these two variables did not differ from unity (Fig. 9). For summer the slope *b* did significantly differ from unity, as reproductive investment decreased with hermaphrodite body mass (Paired *t*-test, *t*(79) = −3.30, *p*-value < 0.001). The primary factor (season) assessed in an ANCOVA, with body mass as the covariate, was significant (*p*-value < 0.001) and showed that there were seasonal differences in the dependent variable (reproductive investment). The interaction term was not significant and therefore was removed and the model re-run (Season: ANOVA, *F*(3,331) = 27.64, *p* < 0.001). After it was re-run, the primary factor season was still significant. Posthoc Tukey multiple comparison tests concluded that reproductive investment in spring was significantly greater than all other seasons and that summer was greater than winter (*p* < 0.05).

**Table 3:** Reproductive investment for hermaphrodites in each season, and regression parameters showing the relationship between hermaphrodite dry body mass and embryo mass after a log-log data transformation.

Season	*n*	R.O. (SD)	Regression parameters		Hypothesis testing		
			Intercept	Slope (SE)	*t*	df	*p*-value
Fall	25	0.15 (0.03)	−0.9049	0.90 (0.18)	−0.22	1.24	0.82
Winter	69	0.14 (0.04)	−0.7466	1.14 (0.12)	−0.64	1.68	0.52
Spring	158	0.20 (0.05)	−0.6895	1.03 (0.10)	−0.181	1.154	0.86
Summer	80	0.17 (0.04)	−0.584	1.21 (0.08)	−3.3	1.79	<0.01*

**Note:**

* Significant at α = 0.05.

## Discussion

The trawl gear overall yielded catches representative of the *L. boggessi* population, despite their small individual sizes, due to debris (i.e., macroalgae, seagrass) reducing pore openings by lining the mesh during tows. Under stress *L. boggessi* were also presumed to seek refuge within this bycatch debris rather than escaping through the net. However, one exception was that this gear type did appear to underrepresent extremely small shrimp with a CL less than three mm. It is possible that these individuals were capable of passing through small openings, or were degraded beyond recognition by debris and biomass in the catch bag. These small shrimp would have been new recruits and likely male phase. *L. boggessi* are also gregarious and are often found cohabiting small crevices in limestone outcroppings (Baeza et al., 2014). Therefore, there was the possibility that the population on The Reef was aggregated at scales smaller than what we were able to measure using the trawl. However, the nocturnal roaming activity of *L. boggessi* (Dickson et al., 2012, unpublished data) was presumed to shift the population more towards a normal distribution during sampling and reduce the potential clustering bias. Although sampling limitations still exist, fisheries dependent sampling via roller-frame trawls is the most effective method for sampling cryptic *L. boggessi*. Thus, this methodology was deemed adequate for our study objectives. The abundance of *L. boggessi* on The Reef was relatively low throughout the year, but peaked in the winter. Sex phase ratios were also male-dominated from fall to early winter and size at sex change decreased dramatically during spring months. Delayed sex change in male shrimp was not directly measured in this study but the similar trends in life history parameters reported here and by Baldwin & Bauer (2003) for *L. wurdemanni* suggest that this phenomenon may also exist for *L. boggessi* on The Reef. Seasonality was also observed in fecundity, reproductive investment and embryo volume.

Even though delayed sex change contradicts the general hypothesis that males should transition into hermaphrodites as soon as possible to increase fitness (Baeza, 2009) this phenomenon may in fact be beneficial if it occurs for *L. boggessi* on The Reef. For instance, it might allow males to capitalize on a sex specific mating advantage, as was observed in *L. wurdemanni* (Bauer, 2002), where large males are more competitive in spawning with post-paturial molt hermaphrodites than small males. However, Baldwin & Bauer (2003) suggested that delayed sex change in temperate *L. wurdemanni* was largely driven by abiotic factors and was a response to suboptimal periods for reproduction. If delayed sex change does occur in *L. boggessi*, then this latter notion is suspected for this population on the west coast of Florida. Similar to the study performed by Baldwin & Bauer (2003) the months where life history characteristics insinuate delayed sex change in *L. boggessi* (high proportions of male phase shrimp and CL_50_ values) coincided with cool temperatures and short photoperiods. These abiotic conditions are presumed to be suboptimal for embryo development and therefore, these males may also have transitioned later to temporarily forego the associated cost of functioning as hermaphrodites (Bauer, 2002, 2005; Baeza, 2006; Zhang, Lin & Lin, 2010). If true, then the advantage of delayed sex change is the ability for males to allocate resources toward growth and survival during suboptimal reproductive periods and then transition into large and more fecund hermaphrodites when conditions improve.

It is also possible that delayed sex change for *L. boggessi* is not adaptive. For instance, assuming that *L*. *boggessi* have short life expectancies (approximately 1 year) similar to *L. wurdemanni* (Baldwin & Bauer, 2003) the high proportion of males during fall may simply be a result of the population consisting of only a few large old individuals and a strong cohort of small young recruits. If true, then the high CL_50_ values during this cool season may be interpreted more as a threshold size at sex change for the species. Additional information on population structure, growth and lifespan is needed to determine the relationships among these factors and life history. Regardless of whether or not delayed sex change is adaptive, there is undoubtedly a strong demographic shift in *L. boggessi* on The Reef from February through May, where the majority of male individuals across all size classes transition into hermaphrodites. This observation supports the notion that PSH *L. boggessi* utilize their sex change plasticity to optimize reproduction and fitness.

The peak reproductive seasons for *L. boggessi* were spring and summer, which were first evident from the high mean fecundity and reproductive investment estimates for individuals during these months (Tables 1 and 3). Although these estimates were greater than for fall and winter, they do not account for the positive correlation between individual size and fecundity and therefore may be misleading if the size structure of the population was different among seasons. However, the ANCOVA regressions (Figs. 7 and 8) did suggest that fecundity for small individuals was equal across all seasons and greater for larger individuals during spring and summer. In addition, spring and summer exhibited lower proportions of males, higher proportions of gravid hermaphrodites and lower CL_50_ estimates than compared to fall and winter. These reproductive characteristics alone do not identify these seasons as being the optimal reproductive period, but in conjunction with the previous life history parameters support this claim.

The results for embryo volume (i.e., greater in winter than summer) were a surprise considering that the high winter estimate (0.15 ± 0.02) more closely resembled the volume for spring than for fall or summer. If winter is truly suboptimal for reproduction, then this finding may be explained by a common tradeoff between fecundity and embryo volume, as reported for other decapod crustaceans (Clarke, 1993; Christiansen & Fenchel, 1979; Hines, 1982; Lardies & Castilla, 2001). For *L. boggessi* this may involve hermaphrodites investing limited resources into a few large embryos vs. numerous small ones, with the rationale that larger embryos contain more nutrients and therefore have a greater probability for individual survival. Alternatively, the winter estimate may be biased due to the categorization of seasons used in this study, considering that the life history parameters (sex ratio and CL_50_) for February appeared to be precursors for a high reproductive period. In addition, there was also a positive correlation between body size and embryo volume during winter (Table 2), which could suggest that the winter estimate is an artifact of the population being skewed towards large individuals.

## Conclusion

Our findings show that the primary breeding seasons for *L. boggessi* on The Reef are spring and summer and high reproductive activity occurs from February to September. We suspect that the seasonality of life history parameters and reproductive characteristics observed here may be, in part, a function of the abiotic conditions (i.e., temperature, photoperiod) at this location. Due to the plasticity of *L. boggessi* size at sex change, seasonality may vary among *L. boggessi* across a latitudinal range, which could have implications for management. To test this hypothesis and to further fill data gaps for fisheries management, similar studies are needed for *L. boggessi* populations in other geographic areas. Future studies should also incorporate methods to assess population size distributions and cohort growth. Lastly, research addressing the influence of biotic factors on *L. boggessi* populations is limited and warrants more attention. For instance, peaks in abundance for *L. boggessi* on The Reef coincided with observed increases in the abundance of *Laurencia* spp. macroalgae. *Laurencia* is ubiquitous throughout Florida waters (Chiappone & Sullivan, 1994), is characterized by high productivity during winter months (Virstein & Carbonara, 1985) and can serve as refugia and transport vehicle for small invertebrates across the benthos (Highsmith, 1985; Trench, 1993; Bell & Hall, 1997; Norrko, Bonsdorff & Norrko, 2000). It is unclear whether *Laurencia* increases the susceptibility to capture for *L. boggessi*, or instead facilitates their immigration from elsewhere. Understanding of the ecology of ornamental species such as *L. boggessi* is equally important as determining the life history and reproductive characteristics for ensuring sustainable harvest.

## Supplemental Information

10.7717/peerj.8231/supp-1 Supplemental Information 1 ANCOVA analyses summarizing the effect of the primary factor season on reproductive output, fecundity and embryo volume. Hermaphrodite body mass and carapace length were controlled for as covariates.

10.7717/peerj.8231/supp-2 Supplemental Information 2 Raw Lysmata Data containing trawl tow information and shrimp sample information.

## Additional Information and Declarations

### Data Availability

The following information was supplied regarding data availability: Raw data is available in the Supplemental Files.
