# Supplementary material for: Life history traits and reproductive performance of the caridean shrimp Lysmata boggessi, a heavily traded invertebrate in the marine aquarium industry
Source: PeerJ. 2020 Jan 23;8:e8231. doi: 10.7717/peerj.8231 (PMC6983297; doi:10.7717/peerj.8231)
Supplement: Supplemental Information 1 [file peerj-08-8231-s001.docx]

**Table** ANCOVA analyses summarizing the effect of the primary factor season on reproductive output, fecundity and embryo volume. Hermaphrodite body mass and carapace length were controlled for as covariates

| ANCOVA (Factors) | df | F-stat | p-value |
| --- | --- | --- | --- |
| Reproductive Output |  |  |  |
| Body Mass | 1 | 7.92 | .005^*^ |
| Season | 3 | 27.79 | < .001^*^ |
| Body Mass x Season | 3 | 1.61 | 0.185 |
| Fecundity |  |  |  |
| Body Mass (covariate) | 1 | 0.41 | 0.518 |
| Season | 3 | 14.1 | < .001^*^ |
| Body Mass x Season | 3 | 5.67 | < .001^*^ |
| Fecundity |  |  |  |
| CL (covariate) | 1 | 1.72 | 0.19 |
| Season | 3 | 14.49 | < .001^*^ |
| CL x Season | 3 | 8.38 | < .001^*^ |
| Embryo Volume |  |  |  |
| Body Mass (covariate) | 1 | 31.06 | < .001^*^ |
| Season | 3 | 43.71 | < .001^*^ |
| Body Mass x Season | 3 | 2.07 | 0.104 |
| Embryo Volume |  |  |  |
| CL (covariate) | 1 | 18.6 | < .001^*^ |
| Season | 3 | 45.11 | < .001^*^ |
| CL x Season | 3 | 1.62 | 0.183 |
| * Significant at α = 0.05 |  |  |  |
